# P-827. Epidemiology of Favorable vs. Unfavorable Outcomes in Hospitalized Patients with *S. aureus* Bacteremia in the US: A Multicenter Retrospective Cohort Study

**DOI:** 10.1093/ofid/ofae631.1019

**Published:** 2025-01-29

**Authors:** Marya D Zilberberg, Brian H Nathanson, Rolf J Wagenaar, Jan Posthumus, Andrew Shorr

**Affiliations:** EviMed Research Group, LLC, Goshen, Massachusetts; OptiStatim LLC, Longmeadow, Massachusetts; Basilea Pharmaceutica International Ltd, Somerville, New Jersey; Basilea Pharmaceutica International., Alschwil Ltd, Allschwil, Basel-Landschaft, Switzerland; Medstar Washington Hospital Center, Not Applicable

## Abstract

**Background:**

The incidence of *Staphylococcus aureus* bacteremia (SAB) remains ∼25 cases/100,000 population, and methicillin-resistant *S. aureus* (MRSA) causes ∼1/2 of all SAB. Historically, mortality has served as the primary gauge for SAB’s outcomes, potentially neglecting other important unfavorable outcomes (UO) that contribute to its morbidity. We developed a multifaceted definition for UOs in SAB and examined their prevalence and early predictors.

**Figure:**
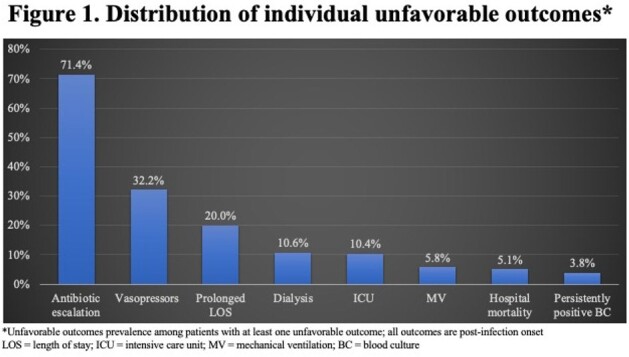

**Methods:**

We included adult hospitalized patients with at least one positive blood culture (BC) for *S. aureus* in a large US database (∼300 hospitals, 2020-2022). We defined UO as at least one of the following: hospital mortality, antibiotic escalation, persistently positive BCs, prolonged post-infection length of stay (LOS), or readmission within 30 days after discharge. All other outcomes were classified as favorable (FO). We used descriptive statistics to compare patients with UO and FO, and applied multiple regression models to identify factors associated with UOs.

**Figure:**
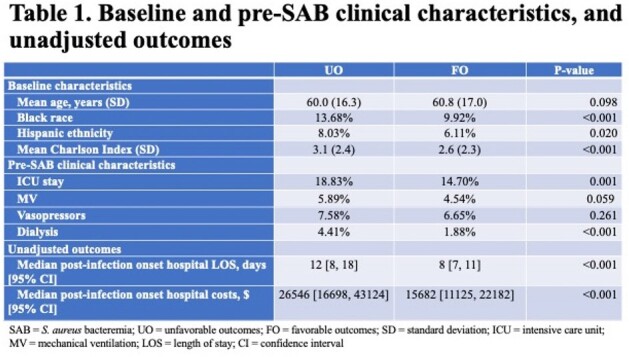

**Results:**

Among 4,080 patients with SAB, 2,427 (59.5%) suffered an UO. The most common UOs were disease worsening and need for vasopressors (Figure 1). Those with UOs were more likely to be non-White and suffered from a higher co-morbidity burden (Table 1). During hospitalization and prior to the onset of SAB, those with UOs more frequently required an ICU stay and dialysis. The majority of SABs were community-onset, and similarly distributed in the groups. UOs occurred in 66.1% of patients with MRSA vs. 55.2% with MSSA. Vancomycin was the leading empiric drug administered, followed by cefazolin. Daptomycin was used infrequently, and the rate of delayed therapy was low. The unadjusted post-infection hospital LOS and costs were significantly higher with UOs (Table 1). The variables with the highest odds ratios predicting an UO were the presence of septic shock on admission, empiric treatment with daptomycin, and the presence of complicated SAB (Table 3).

**Figure:**
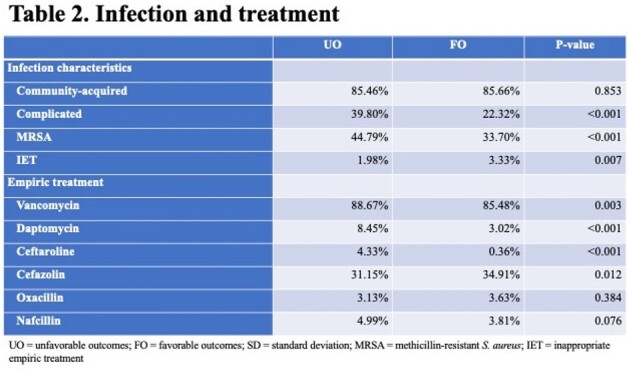

**Conclusion:**

UOs occur frequently in SABs. A broader perspective exploring issues other than mortality demonstrates the substantial implications of SAB both for patients and healthcare systems. Select clinical variables are associated with UOs, some of which may not be modifiable.

**Figure:**
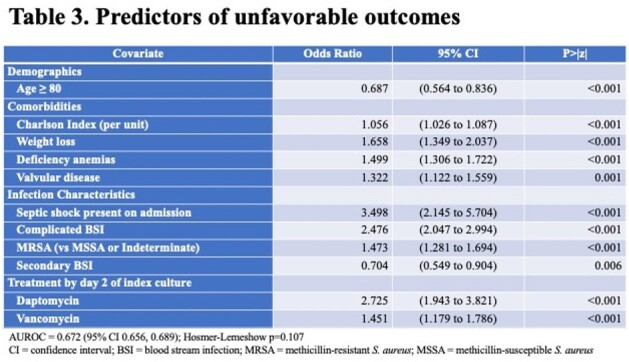

**Disclosures:**

**Marya D. Zilberberg, MD, MPH**, Basilea Pharmaceutica International Ltd, Allschwil, Switzerland: Grant/Research Support **Brian H. Nathanson, Ph.D.**, MERCK: Advisor/Consultant **Rolf J. Wagenaar, MS**, Basilea: Advisor/Consultant **Jan Posthumus, PhD, MBA**, Basilea Pharmaceutica International., Alschwil Ltd: Honoraria|Basilea Pharmaceutica International., Alschwil Ltd: Stocks/Bonds (Public Company) **Andrew Shorr, MD, MPH, MBA**, Basilea: Advisor/Consultant|Basilea: Grant/Research Support

